# Fifty Years of Aflatoxin Research in Qidong, China: A Celebration of Team Science to Improve Public Health

**DOI:** 10.3390/toxins17020079

**Published:** 2025-02-09

**Authors:** Jian-Guo Chen, Yuan-Rong Zhu, Geng-Sun Qian, Jin-Bing Wang, Jian-Hua Lu, Thomas W. Kensler, Lisa P. Jacobson, Alvaro Muñoz, John D. Groopman

**Affiliations:** 1 Department of Epidemiology, Qidong Liver Cancer Institute, Qidong People’s Hospital, Affiliated Qidong Hospital of Nantong University, Qidong 226200, China; yuanrongzhu44@163.com (Y.-R.Z.); wangjinbing826@163.com (J.-B.W.); jianhualu3@163.com (J.-H.L.); 2 Shanghai Cancer Institute, Shanghai Jiaotong University, Shanghai 200032, China; qiangengsuntong@163.com; 3 Department of Environmental Health and Engineering, Johns Hopkins Bloomberg School of Public Health, Baltimore, MD 21205, USA; jgroopma1@jhu.edu; 4 Department of Epidemiology, Johns Hopkins Bloomberg School of Public Health, Baltimore, MD 21205, USA; ljacobs1@jhu.edu (L.P.J.); amunoz@jhu.edu (A.M.)

**Keywords:** aflatoxin B_1_, aflatoxin-N^7^-guanine, aflatoxin-albumin adduct, liver cancer, chemoprevention, clinical trials, oltipraz, chlorophyllin, sulforaphane, broccoli sprouts

## Abstract

The Qidong Liver Cancer Institute (QDLCI) and the Qidong Cancer Registry were established in 1972 with input from doctors, other medical practitioners, and non-medical investigators arriving from urban centers such as Shanghai and Nanjing. Medical teams were established to quantify the extent of primary liver cancer in Qidong, a corn-growing peninsula on the north side of the Yangtze River. High rates of liver cancer were documented and linked to several etiologic agents, including aflatoxins. Local corn, the primary dietary staple, was found to be consistently contaminated with high levels of aflatoxins, and bioassays using this corn established its carcinogenicity in ducks and rats. Observational studies noted a positive association between levels of aflatoxin in corn and incidence of liver cancer across townships. Biomarker studies measuring aflatoxin B_1_ and its metabolite aflatoxin M_1_ in biofluids reflected the exposures. Approaches to decontamination of corn from aflatoxins were also studied. In 1993, investigators from Johns Hopkins University were invited to visit the QDLCI to discuss chemoprevention studies in some townships. A series of placebo-controlled clinical trials were conducted using oltipraz (a repurposed drug), chlorophyllin (an over-the-counter drug), and beverages prepared from 3-day-old broccoli sprouts (rich in the precursor phytochemical for sulforaphane). Modulation of biomarkers of aflatoxin DNA and albumin adducts established proof of principle for the efficacy of these agents in enhancing aflatoxin detoxication. Serendipitously, by 2012, aflatoxin exposures quantified using biomarker measurements documented a many hundred-fold reduction. In turn, the Cancer Registry documents that the age-standardized incidence rate of liver cancer is now 75% lower than that seen in the 1970s. This reduction is seen in Qidongese who have never received the hepatitis B vaccination. Aflatoxin mitigation driven by economic changes switched the dietary staple of contaminated corn to rice coupled with subsequent dietary diversity leading to lower aflatoxin exposures. This 50-year effort to understand the etiology of liver cancer in Qidong provides the strongest evidence for aflatoxin mitigation as a public health strategy for reducing liver cancer burden in exposed, high-risk populations. Also highlighted are the challenges and successes of international team science to solve pressing public health issues.

## 1. Introduction

Primary liver cancer (PLC) is the third leading cause of cancer mortality globally, surpassed only by lung and colorectal cancers. As of 2024, there were over three-fourths of a million deaths world-wide annually from liver cancer, accounting for more than 8% of all cancer deaths [1]. In 2024, in China, PLC was the second leading cause of cancer death with over 316,000 deaths annually [2]. Multiple etiologic factors contribute to the global burden of liver cancer and include infections with hepatitis B (HBV) and/or hepatitis C (HCV) viruses, alcohol, aflatoxins, smoking and air pollution, and diseases associated with disorders in metabolism [3]. It is estimated that there might be up to 5 billion people worldwide who are at risk from aflatoxin exposure, most prominently in countries that have corn- or ground nut-based diets [4]. Epidemiological surveys carried out over the past decades in Asia and Africa have revealed a strong statistical association between aflatoxin ingestion and PLC incidence [5,6]. In China, aflatoxin contamination in food is also widespread. Corn provides more than 50% of the contribution to dietary exposure to aflatoxins in the Chinese population, with higher dietary exposure to aflatoxins in rural than in urban populations [7]. Many of these etiologic risk factors are modifiable, and liver cancer should be largely preventable. For over 50 years, Qidong, China, has been recognized as a hotspot for liver cancer incidence. Moreover, it has been the crucible for studies on etiology and prevention of liver cancer with a strong focus on the role of aflatoxin and its interplay with HBV [8]. As of 2024, over 80% of liver cancer cases in Qidong are in hepatitis B surface antigen positive (HBsAg+) patients despite the implementation of universal vaccination of newborns more than 2 decades ago. However, with a median incident age of 67 years, clearly, almost all cases never received the preventive vaccine. The promise of vaccination will play out in upcoming decades. Yet, the age-standardized incidence of liver cancer in Qidong dropped by about 70% over the 1990 to 2020 period [9]. A profound drop—several hundred-fold—in aflatoxin exposure in this community likely accounts for this remarkable outcome. Aflatoxin research has been a central component of the mission of the Qidong Liver Cancer Institute (QDLCI).

**Figure 1:**
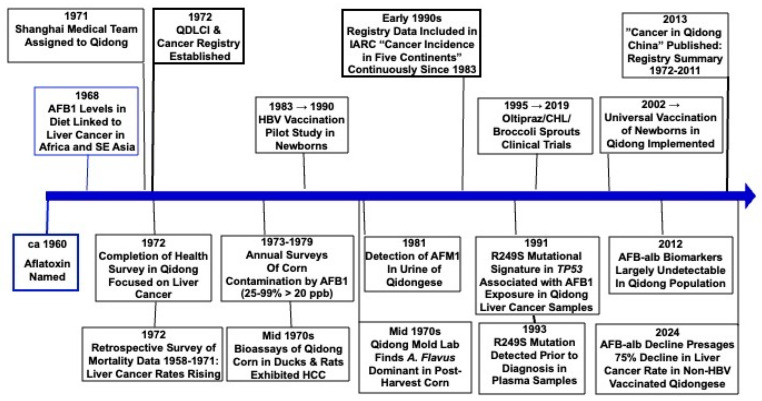
Timeline for the founding and achievements of the Qidong Liver Cancer Institute with regard to their studies on the etiologic role of aflatoxins in the high incidence and mortality of liver cancer in this region. IARC, International Agency for Research on Cancer; AFB-alb, aflatoxin-albumin adduct biomarker; HCC, hepatocellular carcinoma.

This review highlights the seminal contributions of scientists at the QDLCI along with their domestic and international collaborators in defining the importance of aflatoxin exposures in the etiology of liver cancer in this hotspot region of China and the efforts to evaluate strategies to mitigate these exposures and their adverse health impacts. Figure 1 presents a graphical timeline of the key scientific achievements at the QDLCI in this effort and serves as an outline for the review. A goal of this narrative is to indicate some of the challenges and rewards of team science in the pursuit of improving public health.

## 2. A Need for Aflatoxin Research in Qidong, China (1971–1992)

Research on PLC prevention and treatment in Qidong began in 1971 amidst the Cultural Revolution in China. In November 1971, the first group of the “Shanghai Tumor Research Team to Qidong County” (locally known as the “Shanghai Medical Team”) was mandated relocation from Shanghai. The team leader (1971–1973) was Dr. Lu-Yi Yu, former vice president of Shanghai Cancer Hospital. The team consisted of 13 members from various medical and research institutions, including the Shanghai Cancer Hospital, Shanghai Zhongshan Hospital, Shanghai Cancer Institute, and Shanghai First Medical College (now Fudan University Shanghai Medical College), covering disciplines such as internal medicine, gynecology, nursing, laboratory testing, and health statistics. In 1972, Jiangsu Province also sent a medical team to Qidong consisting of members from 12 scientific research and educational institutions, including clinical and preventive medicine, hydrology, soil, geology, geography, and agronomy. Among the Jiangsu Medical Team members were Dr. Yu-Tang Gao from the Department of Medical Statistics at Suzhou Medical College and Dr. Geng-Sun Qian from the Biology Department at Nanjing University (they were later transferred to the Shanghai Cancer Institute, and both later served as directors of that Institute). Their longstanding collaborative relationship with researchers in Qidong enabled international collaborations in later decades.

To detect cancer patients early, under the guidance and participation of the Shanghai and Jiangsu experts, the Qidong Health Bureau organized nearly 700 medical staff into 18 survey teams in early January 1972. Equipped with diagnostic and treatment instruments, they went deep into each village and spent nearly a year conducting a health survey focusing on liver cancer among the population aged 16 and above in Qidong County, covering 580,000 people. This survey provided an accurate understanding of the health status of the population and the prevalence of malignant tumors. In 1972, AFP (alpha-fetoprotein) testing continued, and in May and October, surveys were conducted on the population aged 16 and above and liver disease patients in the Huilong, Xiangyang, and Xining communes (townships), with a total of 71,585 people examined. Among them, 428 tested positive for AFP, and 105 cases of liver cancer were identified [10,11].

At the same time, additional epidemiological and etiological studies on liver cancer were initiated. These included follow-up surveys on patients with hepatitis and cirrhosis discharged from Qidong People’s Hospital since 1964, HBsAg testing, and investigations of the relationship between liver cancer and environmental factors such as water and soil, aflatoxin contamination in food, and family surveys of liver cancer patients.

To understand the trends and distribution characteristics of liver cancer incidence and mortality in Qidong, with support from the local government, broad public cooperation, and guidance from medical teams, a retrospective investigation was organized in July and August 1972. More than 750 medical personnel surveyed cancer mortality data from 1958 to 1971, covering a population of 1.03 million. The investigation involved discussions with elderly farmers, veteran party members, and senior officials, using a retrospective method of all-death causes and calculating the year of death, which helped provide a timeline for determining mortality rates. The results showed that from 1958 to 1971, the overall all-cause death rate in Qidong decreased yearly, from 8.26 to 6.62 per 1000. However, the mortality rate from malignant tumors significantly increased, rising from 56.7 per 100,000 to 124.5 per 100,000. Notably, liver cancer mortality increased substantially, from 20.5 per 100,000 to 49.0 per 100,000. Among males aged 30 to 49, for every four deaths, one was caused by liver cancer, highlighting the severe impact of liver cancer in this region. The investigation also revealed that liver cancer accounted for the highest proportion (35%) of all malignant tumor deaths, followed by stomach cancer at 29% [11,12]. This comprehensive survey confirmed that Qidong was a high-incidence area or hotspot for liver cancer in China.

**Figure 2:**
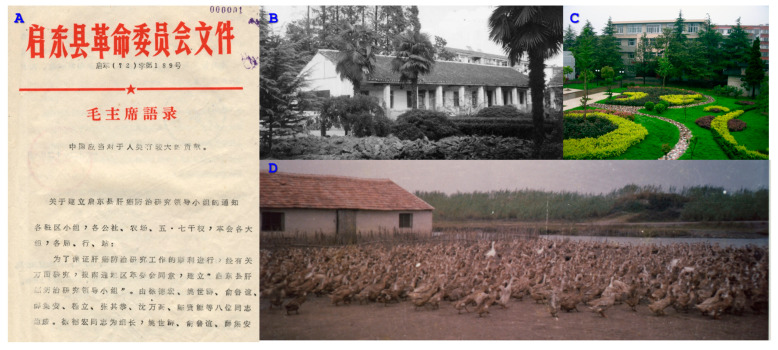
(**A**) Qidong County Government document on the establishment of “Qidong Liver Cancer Research Leadership Group” (1972). Headers in red: “Documents of the Revolutionary Committee of Qidong County DRCQC (1972) No. 189. Quotations from Chairman Mao: China should make a greater contribution to mankind. (**B**) Dormitory and working rooms of “medical team members” in Qidong (early 1970s). (**C**) Research building of the Qidong Liver Cancer Institute (2002). (**D**) Liver Cancer Research Experimental Duck Farm next to the Yellow Sea of Qidong (late 1970s).

In conjunction with the training for the retrospective mortality survey, a county-wide network for cancer control was established, with dedicated or part-time personnel assigned to engage in cancer prevention activities at all levels of medical institutions. A cancer registration and reporting system was also implemented. On 8 December 1972, the Qidong Liver Cancer Control and Research Leadership Group was established. The group was led by the vice governor of the county government, with the deputy director of the Nantong District Health Bureau, the head of the Shanghai Medical Team, and the head of the Jiangsu Medical Team as vice leaders and the director of the Qidong Health Bureau as the office director. This marked the official launch of on-site research on liver cancer prevention and treatment in Qidong and was the foundation for the Qidong Liver Cancer Institute (Figure 2).

***Ecological and case-control studies on aflatoxin contamination in high and lower incidence communes (1970s).***

Multiple food collections were undertaken during the 1970s to measure the extent of aflatoxin contamination of the primary foodstuffs of Qidongese. For example, grain samples were collected in December 1972, May 1973, and September 1973, totaling 711 samples. The results indicated that corn was heavily contaminated by aflatoxin, with 95% of samples showing contamination. *Aspergillus flavus* was identified in most samples; other fungi identified included *Penicillium* and *Aspergillus niger* [13]. The aflatoxin B_1_ (AFB_1_) levels from those collections are shown in Table 1.

A follow-up report showed that the contamination of corn with AFB_1_ was substantial in Qidong from 1973 to 1980 [14]. The contamination rate of corn ranged from a minimum of 26% to a maximum of nearly 99%, with an average of 47% (Table 2). AFB_1_ content greatly exceeded the national permissible standard (20 ppb). These findings indicate a serious problem with AFB_1_ contamination in Qidong, suggesting that the region was a hotspot for aflatoxicosis, with a significantly high number of positive results across various food and grain samples. The contamination of both raw and cooked foods highlighted the serious health risk posed by AFB_1_.

Isolation and culture results of mold from staple foods and grain storage containers in Qidong indicated various levels of *Aspergillus flavus* contamination. Corn was the most severely affected, with a contamination rate of 95%; wheat followed at 19.5%, and polished rice had the lowest at 2%. Grain storage containers, cabinets, dining tables, team warehouses, and food processing areas also showed *Aspergillus flavus* contamination, with an overall contamination rate of 75%.

The QDLCI isolated 4278 *Aspergillus flavus* strains, and 694 (~16%) were found to produce AFB_1_ [13]. This indicated that not only were grains and foods in Qidong contaminated, but the natural environment also harbored significant amounts of *Aspergillus flavus*, with a considerable proportion being toxin-producing strains.

Hot and humid environments are known to foster mold growth in corn and other foodstuffs and feeds. According to the analysis of meteorological data in Qidong in the 1970s, during the corn harvest period every year (August), there was an association between the number of rainy days and the degree of toxin contamination in grain [14].

Residents of communes with a high incidence of PLC were likely to ingest more aflatoxin from corn than those of communes with a low incidence. The contamination rate of AFB_1_ in corn in the Tongxing commune with a high incidence of liver cancer was significantly higher than in the Xining commune with a low incidence of liver cancer in Qidong County. Of the positive corn samples in Tongxing, 64.5% contained more than 20 ppb of the toxin, compared with only 29.2% in Xining [15] (Table 3). Such “geographical pathology” provides another descriptive element of association between agent and disease.

In April 1981, 12 households were randomly selected in each of two communes of Qidong County, Hehe and Xining, and meal samples were collected for three consecutive days for the detection of AFB_1_ and the calculation of the weight of staple foods consumed in 381 samples of cooked staple foods (meals) collected from the two communes. The results indicated a correlation between the detection rate of AFB_1_ in cooked staple foods and the intake of the residents and the mortality rate of liver cancer [16] (Table 4).

According to a survey conducted in 1974 [15], in the communes where the mortality rate of liver cancer was more than 40/100,000, the average percentage of households with more than half of their staple foods as corn was 59.3%. In contrast, in the communes where the mortality rate of liver cancer was less than 25/100,000, the average percentage of households with more than half of their staple foods as corn was only 18.2%. The detection rate of AFB_1_ in cornmeal was 38.1% in communes with a high incidence of liver cancer, whilst the detection rate in communes with a relatively low incidence of liver cancer was 9.3%. The mortality rate of liver cancer was also higher in areas with a large proportion of corn production. The detection rate of AFB_1_ in corn was higher in coastal areas than inland areas, which seemed to be consistent with the geographical distribution of liver cancer.

***Aflatoxin-induced Liver Cancer in Animals: Ducks and Rats (1970s)***

*Field Studies.* In Qidong, poisoning in livestock and poultry due to feeding of moldy corn was frequently observed. This occurred particularly during the corn harvest season in August and September, when prolonged rainy weather led to increased moldy corn. Additionally, spontaneous liver cancer was commonly found in livestock and poultry.

In the Mid-Autumn Festival of 1973, 1321 duck livers were examined, and pathological tests revealed 43 cases of duck liver cancer, with an incidence rate of 3.3% [13,17]. According to the investigation, the feed used for the ducks, which was primarily corn, often contained aflatoxins. Over the years, rats and ducks were fed corn containing aflatoxins, and liver cancer was induced in both. Therefore, the presence of AFB_1_ in Qidong’s corn was likely an important factor in the etiology of human liver cancer. The incidence of liver cancer and cirrhosis in ducks in Qidong was quite high, and the pathological changes suggested a possible link to aflatoxin poisoning. It seemed reasonable to anticipate that the same primary factor responsible for human liver cancer in Qidong was similarly involved in duck liver cancer.

**Figure 3:**
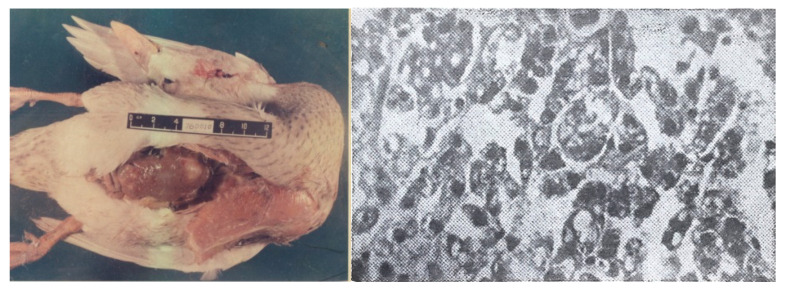
Duck liver cancer and its cytoarchitecture. (**Left**): A mallard duck with liver cancer from a group fed with aflatoxin-containing moldy corn. (**Right**): Duck hepatocellular carcinoma with trabecular arrangement and visible blood vessels.

*Bioassays of Moldy Corn from Qidong.* Several bioassays were conducted using moldy corn from Qidong. In one experiment, mallard ducks were fed corn contaminated with 90 ppb AFB_1_ for 8 days, and after a 2-month interval, they were fed 30 ppb AFB_1_ for a 2-year observation period. Among 75 experimental ducks, 25 developed PLC, resulting in a carcinogenic rate of 33.3%. The control group, fed uncontaminated corn, did not show any abnormalities, a difference that was statistically different [15]. In another experiment, 36 rats were fed corn contaminated with 15–250 ppb AFB_1_ for 2 years (accumulated intake of 1.17–1.32 mg AFB_1_ per rat); 24 of them developed PLC, resulting in a carcinogenic rate of 66.7%. In Yangzhou, which is nearly 250 km from Qidong, ducks were fed contaminated corn from Qidong. Among 50 ducks, 22 developed various tumors, including 12 cases of PLC, resulting in a liver cancer incidence rate of 24.0% [18]. In another study conducted in Yangzhou with moldy Qidong corn over the period of December 1973–June 1976, 80 mallard ducks, 2.5 months old and weighing about 2 kg, were divided into two groups: a moldy corn group (50 ducks) and a control group (30 ducks) [15]. They were kept in enclosures on land, drinking local tap water, and fed a mixture of broken rice, bran, and green fodder. The moldy corn group was fed a mixture containing 150–500 ppb AFB_1_, and the ducks were allowed to consume the food freely. Liver biopsies were conducted periodically, and any deaths were examined with routine histopathology. The ducks were fed moldy corn for a cumulative period of 13 months. In the first 3 months of feeding, 12 ducks died, and all were found to have acute or subacute toxic hepatitis. After 16 months of feeding, tumors began to appear, and by the end of the 29-month experiment, 22 tumors were identified. Among these, 12 were primary liver cancers (5 hepatocellular carcinoma, 4 cholangiocarcinoma, 3 mixed types). In total, 22 ducks with tumors included 1 with three different types of tumors, 9 with two types, and 12 with a single type. The male-to-female ratio for primary liver cancer was 6:6. No tumors were found in the control group, and histological examination of the liver showed no significant lesions. Figure 3 shows a gross specimen of duck hepatocellular carcinoma and cytoarchitecture.

***Determination of Aflatoxins in Human Blood Samples (mid-1970s)***

From November 1975 to April 1976, a total of 524 Qidongese blood samples were tested for AFB_1_ [15]. Seven samples were positive, and follow-up investigations on four of these samples revealed that the AFB_1_ contamination was related to the consumption of food contaminated with AFB_1_ prior to blood collection. After discontinuing the intake of AFB_1_-contaminated food, follow-up blood tests showed negative results, suggesting that the presence of AFB_1_ in the blood may be due to its ingestion through food.

In 1981, a thin-layer chromatographic method for detecting AFB_1_ and AFM_1_ in human urine was established in Qidong. In urine samples from 45 individuals who had ingested moldy corn (AFB_1_ > 20 ppb). AFB_1_ was detected in 2 samples (4.4%), while AFM_1_ was detected in 21 samples (46.7%) [16]. A study on the excretion levels of AFM_1_ in the urine of residents from Beijing and Qidong showed that the 24 h urine AFM_1_ excretion in normal individuals from Beijing was generally below 2 micrograms. In some instances, a significant increase was observed, primarily due to moldy rice consumption. In contrast, the excretion level of AFM_1_ in urine from Qidong was significantly higher. Some individuals in this area had aflatoxin intake levels nearly two orders of magnitude higher than the normal population in Beijing, with an estimated annual cumulative intake exceeding 1 milligram [19,20].

***Prevention and Control of Mycotoxin Contamination (mid to late 1970s)***

**Figure 4:**
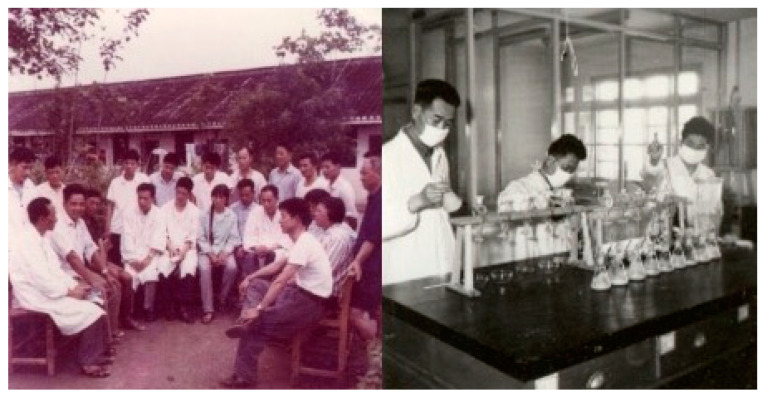
(**Left**). Members of the Medical Team discussing the prevention and treatment of liver cancer with local “Barefoot Doctors” at Jianghai District Hospital, Qidong (1974). (**Right**). Qidong researcher working with the “Medical Team” members at the Qidong Mold Lab (1973). Photos courtesy of Jian-Guo Chen.

Between 1975 and 1977, a three-year study was conducted across six townships in Qidong to track fungal infections in corn at different growth stages. Fungal infections increased as corn matured. The infection rate increased dramatically during the post-harvest dehulling and drying stages, with *Aspergillus flavus* dominating the fungal population. Research suggested that adequate sunlight exposure and maintaining a moisture content below 13% could significantly reduce AFB_1_ contamination during drying [14]. Figure 4 shows Qidong Mold Lab and Medical Team members at work.

Further research into AFB_1_ detection methods led to the development of a portable fluorescence detection device, which was successfully used to survey 2481 corn samples in local communities in Qidong. The device detected a contamination rate of 19.7%, and further analysis using thin-layer chromatography confirmed that 70% of the fluorescent-positive samples had AFB_1_ levels exceeding 20 ppb. The use of this detection method greatly improved the efficiency of investigating and controlling aflatoxin contamination in local food sources [14].

Additionally, anti-fungal measures to reduce fungal contamination and aflatoxin production were tested in Qidong. Early research in China showed that sodium metabisulfite was highly effective in preventing mold on wet corn, and ethylene oxide was found to be the most effective agent for preventing rice spoilage. Chemical detoxification methods, including sodium carbonate, chlorine, and lime water treatments, were tested for neutralizing AFB_1_. Sodium carbonate proved to be the most effective, reducing aflatoxin levels to undetectable levels after 5 to 15 min of exposure. Chlorine treatment produced potentially harmful chlorinated proteins, limiting its practical application. Experiments on the production of starch and alcohol from aflatoxin-contaminated corn demonstrated that these processing methods could reduce AFB_1_ to undetectable levels [21]. However, although these chemical methods are effective in decontaminating moldy corn, the possible toxic effects of adding chemicals to the food could not be ruled out, and this, together with the fact that the chemical decontamination methods affected the appearance of the grain and produced an off flavor, made them unsuitable for generalized promotion, and they were not adopted in the field. Nonetheless, this research emphasized the importance of early intervention during storage to prevent fungal contamination.

Studies on detoxification explored other various methods, including “sorting, peeling and degermination” and chemical treatments. The sorting method involved manually removing visibly contaminated corn kernels and proved effective for mildly contaminated samples and reducing AFB_1_ levels. Peeling and degermination, especially wet peeling, also effectively reduced AFB_1_ concentrations to below the national safety limit of 20 ppb. However, degermination resulted in significant nutritional losses, as most AFB_1_ is concentrated in the germ [21].

In conclusion, the findings from Qidong in the 1970s and early 1980s provided strong evidence that aflatoxin contamination in food sources, particularly corn, played a significant role in the high incidence of liver cancer in the region. While these studies shared the weaknesses of ecological studies in establishing causality, they underscored the necessity of increased public awareness and control measures to limit aflatoxin exposure. The correlation between animal models and human liver cancer incidence suggested that aflatoxin contamination in staple foods posed and continues to pose a severe public health risk, necessitating targeted intervention strategies in affected regions.

***Qidong Cancer Registry (founded 1972)***

The fourteen-year retrospective mortality survey (1958–1971) initiated in 1972 provided valuable scientific evidence to understand the prevalence of malignant tumors in the Qidong area and to guide future research directions. This investigation not only revealed the severity of liver cancer but also underscored the growing importance of disease surveillance. A single retrospective survey reflects only past conditions, and to achieve long-term goals in cancer prevention and treatment, it was essential to establish a sustainable and effective registration and reporting system. This system would facilitate tracking of incidence trends and provide reliable data to inform national policies on cancer prevention and control.

There was a consensus on the need to establish a regular malignant tumor registration and reporting system in Qidong. Firstly, the local health prevention network in Qidong was well-established, with health centers in every township and basic medical personnel at the village level, providing robust organizational support for data collection and reporting. Additionally, China’s success in cancer registry work in Shanghai at that time served as a direct point of reference. Secondly, the experts from the Shanghai Cancer Institute (Shanghai Cancer Registry), Shanghai First Medical College, and Nanjing Medical College in epidemiology and medical statistics were also members of the “Medical Team” and played a crucial role in the establishment and operation of the Qidong Cancer Registry.

Qidong assigned a dedicated team of personnel at the village, town, district, and county levels to be responsible for case ascertainment, reporting, registration, and data management. Therefore, since the initiation of the project in 1972, the Qidong Cancer Registry has, through practical experience, adapted and optimized the methods and data classification standards from the Shanghai Cancer Registry, tailoring them to the characteristics of rural Qidong. This has resulted in the creation of a unique cancer registration and reporting system that continues to be in use today. It is the third oldest cancer registry in China and was the first established in a rural area.

In the early 1990s, Dr. Yu-Tang Gao from the Shanghai Cancer Institute (Shanghai Cancer Registry) recommended the submission of Qidong’s cancer registration data to the International Association of Cancer Registries (IACR), leading to Qidong becoming a unit member of IACR. Starting from Volume VI of the “Cancer Incidence in Five Continents” (CI5), published by the IACR and the IARC (International Agency for Research on Cancer), the cancer incidence and mortality data from Qidong for the period 1983–1987 and subsequent five-year periods have been included in the subsequent volumes of CI5 [22,23].

The continuous cancer registration data from Qidong for over more than 50 years has laid a solid foundation for the epidemiological, etiological, and clinical studies of liver cancer and other cancers and for the evaluation of the effectiveness of prevention measures in Qidong. As such, it has uniquely charted the impact of social, economic and environmental changes in cancer rates.

## 3. The Genesis of an International Collaboration Between the QDLCI and Johns Hopkins University (1993–2025)

Connections between scientists can be purposeful, serendipitous, or a combination of both. In the early 1980s, Dr. Yu-Tang Gao of the Shanghai Cancer Institute and Dr. Brian Henderson at USC were developing plans for establishing a large cancer cohort in men in Shanghai that would be linked to the Shanghai Cancer Registry. The initial focus was on lung and liver cancer. Drs. Gerald Wogan and John Groopman (a former doctoral student of Wogan) were invited to Shanghai in 1985 to discuss including their new biomarker, urinary aflatoxin-N^7^-guanine, into the study design. There, Groopman met Dr. Geng-Sun Qian, leader of the Carcinogenesis Program of the Shanghai Cancer Institute. Henderson and Wogan had met in the early 1980s on seminar tours of American scientists in China, and Wogan and Qian met when the latter was on a sabbatical in 1980 at the US FDA to learn thin-layer chromatography methods for quantitation of aflatoxin contamination [24].

A total of 18,244 men aged 45–64 years living in four small geographically distinct areas of metropolitan Shanghai were recruited into the cohort between 1986 and 1989. Participants completed questionnaires related to diet and other lifestyle factors as well as health history. By the time of a follow-up in 1990, 22 cases of PLC had been identified within the cohort and were matched to 140 controls. Analyses of biomarkers of infection with HBV (HBsAg+) and aflatoxin (AFB-N^7^-guanine) showed a synergy between HBV infection and exposure to aflatoxin, together increasing the risk of liver cancer by greater than eightfold relative to HBV alone [25,26]. The clear message from this landmark study was that elimination of either risk factor could greatly reduce liver cancer incidence in this region. In 1993, Dr. Qian invited Drs. Groopman and Thomas Kensler (another former doctoral student of Wogan), both at Johns Hopkins University (JHU), to meet the leaders of the QDLCI to discuss collaborative ecological and interventional studies targeting liver cancer prevention. As discussed earlier, close ties between the leaders of the Shanghai Cancer Institute and the QDLCI were fostered during the work period of the “Medical Team to Qidong” in the early 1970s.

**Figure 5:**
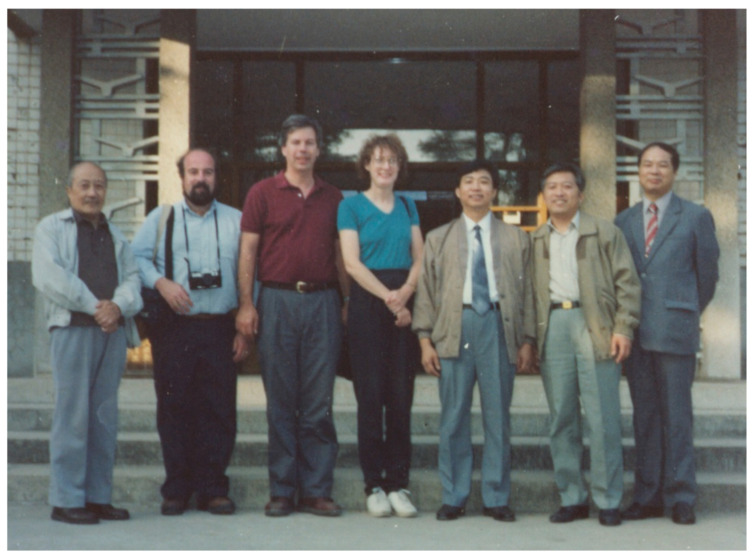
Visit of Johns Hopkins scientists to the Qidong Liver Cancer Institute (September 1993). (**Left** to **right:**) Drs. Lu-Yi Yu, John Groopman, Thomas Kensler, Nancy Davidson, Yuan-Rong Zhu, Geng-Sun Qian, and Bao-Chu Zhang. Photo credit: Jian-Guo Chen.

As a prelude to these discussions, two Hopkins faculty, Drs. Audrey Zarba and Lisa Jacobson, traveled to Qidong in July 1993. Accompanied by Drs. Yuan-Rong Zhu and Jian-Guo Chen, they made a site visit to several villages at Daxin (now spelled Daxing) to examine the feasibility of conducting a small, inexpensive pilot field study to measure current levels of aflatoxin exposures using a newly developed exposure biomarker, aflatoxin-albumin adducts. Thereafter, Drs. Groopman, Kensler, and Davidson, a Hopkins oncologist, traveled to Qidong to formalize the pilot study and explore longer-term opportunities for collaborations in cancer prevention (Figure 5).

The pilot study was conducted in the township of Daxin. It comprised two waves, the first from September–December 1993 and the second from June–September 1994, post- and pre-harvest, respectively, for the annual local corn crop [27]. From 120 consented individuals (men and women), 116 completed the first wave and provided blood samples every 2 weeks, whilst 103 completed the second wave and provided blood samples monthly. Using linear regression models, the mean aflatoxin-albumin adduct levels increased (*p* < 0.05) during the 12 weeks of wave 1 and decreased (*p* < 0.05) over the 4 months of wave 2. The duration of the waves was chosen to encompass several half-lives of circulating albumin (~30 days). Neither HBV surface antigen status nor gender modified either the baseline means or the temporal trends. From the 792 collected serum samples, only one sample had an undetectable level of the aflatoxin-albumin adduct. Buoyed by the consistent and high exposures to aflatoxin, enthusiasm was strong to develop interventional studies.

However, an important observation indicated that no tracking was observed. Thus, while the aflatoxin-albumin adduct demonstrated seasonal variations in population-level exposures, it provided no insight into the trajectories of individual exposure. Subsequent modeling in an aflatoxin hepatocarcinogenesis study in rats similarly indicated that a chemopreventive intervention with oltipraz dramatically reduced tumor incidence and areas under the curve for aflatoxin-albumin adduct levels but only related to protection at the population level and not the individual level [28]. Thus, duration and frequency of sample collections needed to be considered carefully in any future prevention clinical trials.

***Mutational Signatures (early 1990s)***

In the early 1990s, there was an effort to identify molecular signatures, in the form of mutations, in populations with high exposures to environmental carcinogens, aflatoxin foremost among them. Mutational signatures within cancer cells provide new insights into the causes of individual cancers, fingerprinting both endogenous and exogenous factors that influence their development. Whole genome sequencing now enables the detection of thousands of mutations in many forms of cancer [29]. The studies beginning in the 1990s used specific gene sequencing to identify a R249S mutational signature in the tumor suppressor gene *TP53* that was strongly associated with aflatoxin exposure in Qidong samples [30,31,32,33]. The mutation rate of codon 249 of *TP53* in hepatocellular carcinoma specimens collected in 1994–1997 in Qidong averaged 53.6% (52/97), which was significantly higher than that in Beijing (0/22) [33]. Further reflecting an environmental etiology, few mutations in codon 249 have been found in liver cancer from Japan and other areas where there has been little exposure to aflatoxin [34]. Initially, we evaluated genetic alterations in 24 liver resection specimens from Shanghai and Qidong [32]. HBV was integrated in all patient samples. Alterations of *TP53* were present in 95% of the cases. All seven liver cancers from Qidong and three out of five from Shanghai had the aflatoxin-associated point mutation with a G to T transversion at codon 249 (R249S). Similarly, Szymañska et al. [35] detected R249S mutations in 11 out of 16 (64%) liver cancers from Qidong. In a follow-up study utilizing serial samples from a longitudinal collection of plasma samples from a cohort of 1638 high-risk individuals in Qidong, we compared results from plasma DNA and DNA sequencing for specific mutations in 25 liver tumors [36]. Mutations were detected in 10 samples. Analysis of 20 additional plasma-tumor pairs showed that 11 tumors and 6 plasma samples contained the specific mutation. Ten plasma samples from healthy individuals were all negative. Jackson et al. [37] further explored the temporality of detection of this mutation in plasma before and after clinical diagnosis of liver cancer in the same patient. Sixteen patients diagnosed with liver cancer between 1997 and 2001, with plasma samples collected before and after liver cancer diagnosis, were selected for the study. In samples collected prior to liver cancer diagnosis, 22% of the plasma samples had detectable levels of the codon 249 mutation. The codon 249 mutation was detected in 45% of all plasma samples following diagnosis of cancer. Further, persistence of this mutation in plasma once it became measurable was statistically significant in repetitive samples following diagnosis. Nearly one half of the patients were positive for this marker at least 1 year, and in one case 5 years, prior to diagnosis. Huang et al. [38] reported that the R249S mutation in *TP53* was detected at a much higher frequency in the plasma of liver cancer patients from Qidong than in specimens from cirrhotic or healthy controls also residing in Qidong. Subsequently, many studies on liver cancer mutations in populations exposed to high levels of dietary aflatoxin have found high frequencies of G-C to T-A transversions, with clustering at codon 249. The mutational signature “24” is now linked to this aflatoxin specific mutation in hepatocellular carcinoma [39].

Whole genome sequencing in recent years has provided new insights into the contribution of mutagenic exposures to liver cancer development. Zhang et al. [40] sequenced 49 liver tumors collected between 1990 and 2016 in Qidong to refine genetic features associated with aflatoxin exposure. The dominant pattern was G to T transversions. The *TP53* mutation frequency was very high (81.6%), and the major genotype was the R249S hotspot mutation. Additional genes harboring mutations included *TERT*, *AXIN1*, *CTNNB1*, and *ADGRB1*. In this study, the aflatoxin mutational signature, highly represented in the Qidong samples, was observed at low frequencies in tumors from the US (3.5%) and France (1.7%).

In addition, non-target genes (e.g., hypoxanthine guanine phosphoribosyltransferase gene (*HPRT*)) and effector genes (e.g., *HBV* viral genome) have been interrogated. In an early study, we determined somatic mutation frequencies in human *HPRT* [41]. Ninety healthy subjects from Daxin were assigned as having low or high exposure to AFB_1_, according to a dichotomization of their levels of aflatoxin-albumin adducts around the population mean. *HPRT* mutant frequency was determined in individuals by a T cell clonal assay. An odds ratio (OR) of 19.3 (95%CI: 2.0~183) was demonstrated for a high *HPRT* mutation frequency in individuals with high aflatoxin exposure compared with those with low aflatoxin exposure. Several mutations in the *HBV* genome have been identified as well in samples from Qidong and elsewhere [42,43,44,45]. Some may hinder immune recognition and clearance of the virus from the host [46]. However, there is little evidence linking the mutations directly to aflatoxin exposure; rather, they likely reflect the consequences of endogenous mutagenic processes.

***Chemoprevention Trials (1993–2019)***

In the decade of the 1990s, the downward inflection in liver rates was unrecognizable, and strategies for eliminating aflatoxin from the contaminated corn were elusive. Chemoprevention using agents known to enhance the detoxication of aflatoxins in vivo, reduce the burden of DNA damage to the liver, and evoke remarkable efficacy towards preventing liver cancer in animal models offered a potential means for risk reduction in this and other highly exposed populations. A series of clinical trials with a repurposed drug, an over-the-counter medicine, and a widely consumed food ensued. The development of clinical trial protocols for chemoprevention studies in Qidong required considerable reconciliation of Western and Eastern understanding of differences in cultures, trial designs, and governmental regulations (IRBs, INDs, import and export of supplies, agents, and biospecimens). First and foremost, we would utilize a synchronous timeline such that all participants would experience each milestone on the same day. This approach would require a large team to enact it—one not achievable in the West. Participants would also be recruited from a constrained geographical area, typically a single township, for logistical ease and to preserve the notion that all participants shared common features of environmental exposures, such as to aflatoxins.

Most days, these trials ran smoothly in this format, but not all days: typhoons, floods, power failures, and social-political events (chatter amongst neighbor participants; the accidental bombing of the Chinese embassy in Belgrade by NATO forces) created challenging but surmountable obstacles.

*Oltipraz.* The strategy the team identified for the initial clinical disruption of aflatoxin carcinogenesis in Qidong was to attempt to enhance the detoxication of aflatoxin in exposed individuals. Oltipraz, a substituted 1-2-dithiole-3-thione, was originally developed by the pharmaceutical industry as a possible treatment for schistosomiasis and was extensively evaluated in clinical trials in the early 1980s. While studying mechanisms of antischistosomiasis by oltipraz, Bueding and colleagues at Hopkins initially noted that giving the drug to mice markedly elevated glutathione levels in many tissues [47] as well as enzymes important to carcinogen detoxication in multiple tissues [48,49]. These results prompted Bueding to predict that oltipraz might have cancer chemopreventive properties. A lifetime bioassay testing the efficacy of oltipraz against aflatoxin hepatocarcinogenesis in male F344 rats provided a strong justification for its use in Qidong [50]. Additionally, in this study, oltipraz co-treatment led to a 67% reduction in the urinary elimination of aflatoxin N^7^-guanine during the dosing period, indicating that protection afforded by oltipraz presumably results from the marked decrease in levels of hepatic DNA adducts. Extensive preclinical evaluation by the National Cancer Institute (NCI) showed that oltipraz was an effective anticarcinogen in nearly a score of animal models [51].

Supported by the US NCI, a Phase IIa chemoprevention trial of oltipraz was conducted in Daxin township, Qidong, in the summer of 1995 [52,53]. This was a placebo-controlled, double-blind study in which 234 participants were randomized to receive placebo or 125 mg oltipraz daily or 500 mg oltipraz weekly for 8 weeks. Aflatoxin biomarkers [54] and metabolites [55] were used as the primary, albeit intermediary study endpoints. Compliance amongst the study participants was very good [53]. Urinary AFM_1_ levels were reduced by 51% compared with the placebo group in persons receiving the 500 mg weekly dose. No significant differences were seen in urinary AFM_1_ levels in the 125 mg group compared with the placebo. This effect was thought to be due to inhibition of cytochrome P450 1A2 activity by the higher dose only. Median levels of aflatoxin-mercapturic acid (a glutathione conjugate derivative) were elevated sixfold in the 125 mg group but were unchanged in the 500 mg group. Increased aflatoxin-mercapturic acid reflects induction of aflatoxin conjugation through the actions of glutathione transferases. The apparent lack of induction in the 500 mg group probably reflects masking caused by diminished aflatoxin-8-9-epoxide formation for conjugation through the inhibition of CYP1A2 seen in this group [56]. (A full discussion of aflatoxin metabolism is provided in a companion article in this special issue [55].) Aflatoxin albumin adducts were measured in serum samples collected weekly. Individuals receiving 500 mg weekly but not 125 mg daily for 8 weeks showed a triphasic response to oltipraz. No effect was observed during the first month of the intervention, whereas a significant (*p* = 0.001) diminution in adduct levels was observed during the second month of active intervention and during the first month of follow-up. A partial rebound in adduct levels toward baseline values was observed during the second month postintervention. Linear regression models up to week 13 confirmed a significant (*p* = 0.008) weekly decline of biomarker levels in the group receiving 500 mg of oltipraz once a week [57]. This initial study demonstrated for the first time that aflatoxin biomarkers could be modulated in humans.

Fifty-one participants (21.8%) reported adverse clinical events [53]. An extremity syndrome, developing soon after the start of treatment, was the only event that occurred more frequently (*p* = 0.002) among the active groups (18.4 and 14.1% of the daily 125 and weekly 500 mg arms, respectively) compared with the placebo (2.5%). The oltipraz arms did not differ in symptom type or severity. Further clinical evaluation of oltipraz ended several years later when the drug went off patent and synthesis of more drug proved to be too expensive relative to other agents in the NCI pipeline.

**Figure 6:**
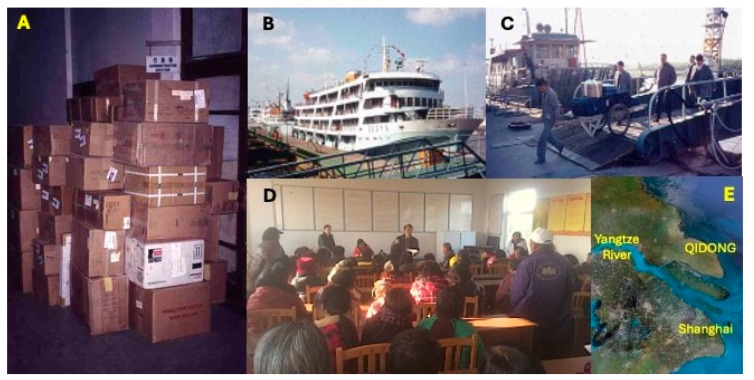
Transport of clinical trial materials to Qidong by ferry (1995). (**A**) Supplies initially shipped to the Shanghai Cancer Institute. (**B**) Overnight ferry from Shanghai to Qidong. (**C**) Off-loading supplies by handcart. (**D**) One of many community meetings led by Dr. Chen to explain the clinical trial and recruit participants for the screening phase of the study. (**E**) Satellite view of the Yangtze River Basin area of China. Photo credits: Thomas Kensler.

**Figure 7:**
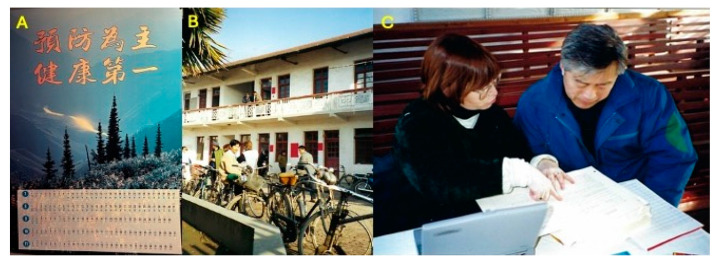
Oltipraz Chemoprevention Trial (1995). (**A**) ”Prevention is Best, Health First” (”yùfáng wéi zhǔ, jiànkāng dì yī”) calendar presenting the synchronous structure of the trial for participants. (**B**) The Daxin Medical Clinical where screening and follow-up were conducted. (**C**) Drs. Jacobson and Qian reviewing eligibility questionnaires. Photo credits: Thomas Kensler.

**Figure 8:**
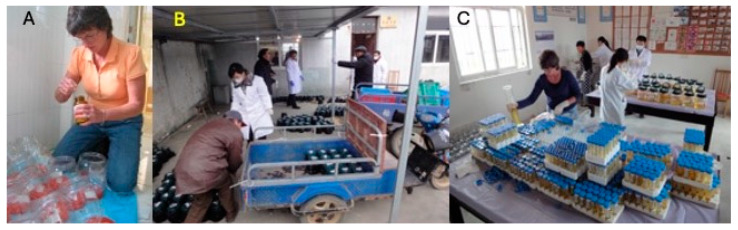
Collecting and processing overnight urine samples for biomarker analyses. (**A**) Adding ascorbate to urine collection containers. (**B**) Receiving and organizing overnight voids from study participants. (**C**) Measuring urine volumes and aliquoting samples for freeze-down at the local ”urinarium.” Photo credits: Thomas Kensler.

The success of this trial was a direct result of the seamless integration of the complementary expertise of the QDLCI and JHU team members. Beyond fulfilling binational regulatory requirements, delivery of clinical trial supplies, including oltipraz tablets formulated by the NCI, and recruitment of study participants in Daxin for screening and possible eligibility were key first steps (Figure 6). Screening included medical histories, blood and urine chemistry, β-scans, EKGs, and physical exams. Because of the synchronous design of the trial, selected participants were provided with a study calendar to highlight days for taking the study drug, providing blood and urine samples, and follow-up clinic visits (Figure 7). Overnight (12 h) urine samples were collected at participants’ homes and quickly processed at a local “urinarium” for subsequent clinical chemistry and biomarker analyses (Figure 8).

*Chlorophyllin.* Chlorophyllins are semi-synthetic metal coordination complexes derived from chlorophyll. Magnesium as the metal coordination element is replaced, and the phytyl chains are removed to create water-soluble salts. A sodium-copper complex (CHL) has long been used as an over-the-counter drug for odor control in ostomy patients and to promote wound healing. CHL is also used as a food colorant. Drs. Bailey and Dashwood and colleagues at Oregon State University defined the actions of CHL as a cancer chemopreventive agent. In animal models of AFB_1_-induced liver cancer, administration of CHL at the same time as dietary AFB_1_ exposure significantly reduced AFB_1_-induced DNA damage in the livers of rainbow trout and rats [58,59] and dose-dependently inhibited the development of liver cancer in trout [60]. Although the primary mode of action is thought to be the sequestration of aflatoxin by chlorophyllin in the gastrointestinal tract [61,62], thereby impeding absorption, we have characterized enzyme-inducing properties, as seen with oltipraz, that may also contribute to its mechanism of action [63]. The OSU investigators conducted an unblinded crossover study of the possible effects of CHL on AFB_1_ pharmacokinetic parameters among a small number of human volunteers [64]. Fasting subjects received an Institutional Review Board-approved dose of ^14^C-AFB_1_ (30 ng, 5 nCi) by capsule with 100 mL water, followed by normal eating and drinking after 2 h. Blood and cumulative urine samples were collected over 72 h, and ^14^C- AFB_1_ equivalents were determined by accelerator mass spectrometry. The study revealed rapid human AFB_1_ uptake and urinary elimination kinetics. CHL treatment significantly impeded AFB_1_ absorption and reduced C_max_ and AUCs (plasma and urine) in the subjects, suggesting that CHL co-consumption may limit the bioavailability of ingested aflatoxin in humans, as they do in animal models. More broadly, this study established that microdosing studies with carcinogens have the potential to provide important insights into chemopreventive interventions and to enhance the overall clinical development and safety evaluation of preventive agents [65].

In a randomized, double-blind, placebo-controlled chemoprevention trial conducted in other Daxin villages in 1997, CHL was determined to alter the disposition of aflatoxin [66]. One hundred and eighty healthy adults were randomly assigned to ingest 100 mg of CHL (provided as Derafil^®^) or a placebo three times a day prior to each meal for 4 months. The primary endpoint was modulation of levels of urinary aflatoxin N^7^-guanine adducts collected three months into the intervention. Adherence to the study protocol was outstanding, and no adverse events were reported. However, both serum and feces were noted to be tinted green [67]. Aflatoxin N^7^-guanine was detected in 105 of 169 urine samples available from this timepoint. CHL consumption at each meal led to an overall 55% reduction (*p* = 0.036) in median urinary levels of this aflatoxin DNA damage biomarker compared with the levels in those subjects taking the placebo [66].

While proof of principle for the interceptor strategy to dampen aflatoxin bioavailability with CHL was clearly established, the practical implementation of population-scale interventions requiring thrice-daily administration potentially for decades precluded further development of this approach. While no concerns about toxicities from chronic administration of CHL have been reported, the potential for collateral increased uptake of copper or diminished uptake of some nutrients would require investigation.

*Broccoli Sprouts (Sulforaphane).* Although the initial oltipraz clinical trial demonstrated the proof of principle for inducing pathways leading to enhanced aflatoxin detoxication in humans, the practicality of using a drug-based method for prevention in the economically developing world is limited; instead, approaches for “frugal medicine” are needed [68]. Many foods have high levels of enzyme inducers [69] that could potentially enhance the detoxication of AFB_1_. In the early 1990s, Dr. Paul Talalay and colleagues at JHU isolated and characterized sulforaphane from broccoli as the most potent naturally occurring inducer found anywhere in the plant kingdom [70]. In the plant, sulforaphane is formed from the precursor glucoraphanin following its hydrolysis by the plant enzyme myrosinase. This group subsequently demonstrated that sulforaphane was an effective chemopreventive agent in animals [71,72]. They further characterized the pharmacokinetics of sulforaphane administered as a broccoli homogenate in human volunteers [73,74].

**Figure 9:**
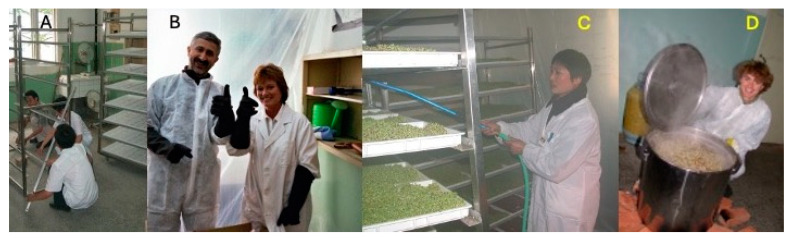
Preparing glucoraphanin-rich broccoli sprout beverage at the Qidong Liver Cancer Institute. (**A**) Building sprouting racks. (**B**) Cleaning the ”greenhouse”: Jed Fahey and Patricia Egner. (**C**) Watering the sprouts every other hour for 3 days. (**D**) Boiling 3-day-old sprouts to make the beverage: Justin Fahey. The first brew was the treatment beverage; a fourth brew of the broccoli mash became the placebo. There was no glucoraphanin (or other inducer activity) in the placebo. Photo credits: Thomas Kensler.

Importantly, they recognized that young plants, as opposed to mature market stage broccoli, held the highest concentration of glucoraphanin by weight [75]. Partnering with Drs. Talalay and Fahey, we developed the first Phase II trial using broccoli sprouts. To provide a consistent dose, a beverage formed from hot water extract of 3-day-old broccoli sprouts, containing defined concentrations of glucosinolates as a stable precursor of the anticarcinogen sulforaphane, was evaluated for its ability to alter the disposition of aflatoxin in a clinical trial conducted in HeZuo, Qidong, in 2003. Twenty kilograms of broccoli seeds (*Brassica oleracea* L., Italica Group) were imported, requiring a long paper trail of chops and approvals. A temporary sprouting facility was built in a QDLCI lab, and over 100 L each of glucoraphanin-rich (GRR) and placebo beverages were prepared, tittered for glucoraphanin levels, aliquoted, and frozen prior to daily administration to study participants (Figure 9).

Two hundred healthy adults drank beverages containing either 400 or <3 μmol glucoraphanin nightly for 2 weeks. At the end of the study, urinary levels of aflatoxin N^7^-guanine were slightly lower in the GRR intervention arms. However, measurement of urinary levels of sulforaphane metabolites indicated striking inter-individual differences in bioavailability. This outcome likely reflected individual differences in the rates of hydrolysis of glucoraphanin to sulforaphane by the intestinal microflora of the study participants (the plant myrosinase was destroyed when the sprouts were boiled in water to prepare the GRR beverage). Accounting for this variability, a significant inverse association was observed for excretion of total sulforaphane metabolites and aflatoxin N^7^-guanine adducts in individuals receiving broccoli sprout glucosinolates [76]. Those individuals exhibiting good bioavailability of sulforaphane had lower levels of aflatoxin biomarkers. This preliminary study illustrated the potential use of an inexpensive, easily implemented, food-based method for prevention. However, despite improvements in the rigor and sensitivity of the analytical methods used to measure the aflatoxin biomarker, it became apparent that aflatoxin exposures were dropping—substantially. The proportion of undetectable samples was increasing.

This study spawned a series of follow-up broccoli sprout-based clinical trials in Qidong and globally in a variety of health maintenance and disease prevention settings. As of 2024, well over 100 broccoli-based clinical trials are listed in ClinicalTrials.gov. Several subsequent trials with broccoli sprout preparations were conducted in Qidong from 2009 to 2019 with an objective of improving the extent and consistency of the bioavailablity of sulforaphane as well as the organoleptic properties—principally taste—of the administered beverages [75,76,77,78,79,80,81]. Because dietary exposures to aflatoxins had been dropping in the 1990s–2000s, it was no longer feasible, nor warranted (*infra*), to use aflatoxin biomarkers as study endpoints. Over the past 20 years, the primary environmental concern in the region had become air pollution. A summary of the randomized clinical trials in Qidong employing aflatoxin biomarkers is provided in Table 5

The chemoprevention trials conducted in Qidong clearly established proof of principle for the strategy of enhancing aflatoxin elimination from humans. While there remains great promise for the use of chemoprevention and interception in some high-risk cohorts [84], perhaps application to settings of heterogeneous exposures to a dietary carcinogen lacks sufficient precision for defining the target cohort. Other prevention modalities may have better cost-benefit and risk-benefit profiles.

***Qidong Cancer Registry: Falling and Rising Cancer Rates (1972–2025)***

**Figure 10:**
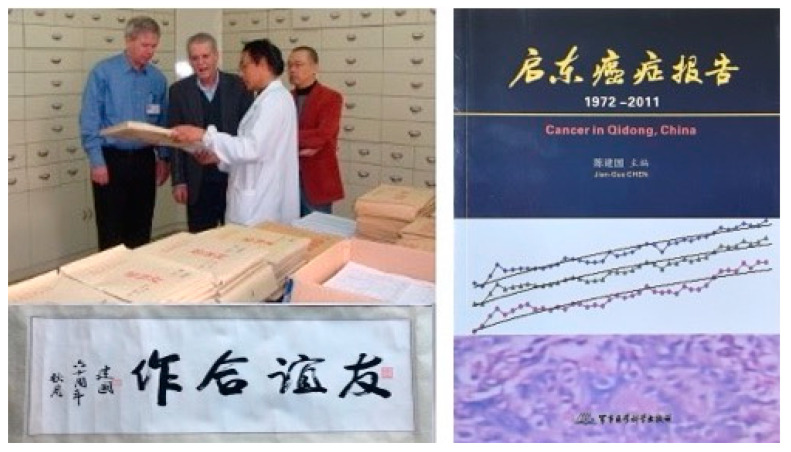
(**Top left**) Early visit to the Qidong Cancer Registry by Dr. Alvaro Muñoz with Drs. Kensler, Jian-Guo Chen, and Tao-Yang Chen (**Bottom left**). Caligraphy for “Friendship Cooperation” (“Yŏuyì Hézuò”) by J-G Chen—Hézuò is also the township where the first broccoli sprout beverage trial was conducted. (**Right**) “Cancer in Qidong, China”, a comprehensive summary of the Qidong Cancer Registry 1972–2011 edited by Jian-Guo Chen [11]. Photo credits: Thomas Kensler.

**Figure 11:**
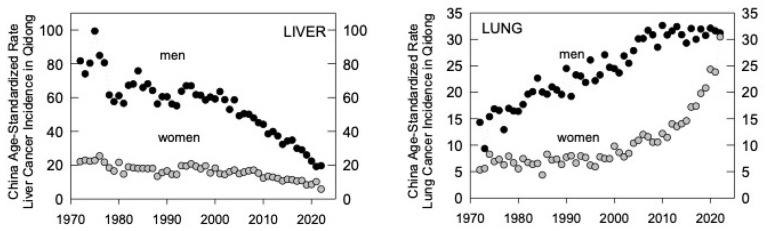
Fifty-year tracking of the age standardized rates for liver and lung cancer incidence in Qidong by gender. Data from the Qidong Cancer Registry.

As the third oldest cancer registry in China and the first established in a rural area, this registry has uniquely tracked the effects of social, economic, and environmental changes on cancer rates over the past 50+ years. Originally paper-based (Figure 10), the registry is now digital, allowing for comprehensive longitudinal analyses of cancer rates [11]. The magnitude of some changes is remarkable, as with liver and lung cancers (Figure 11), and provides powerful insights into underlying etiologies [84,85,86].

The age-standardized incidence of liver cancer in Qidong has dropped remarkably since the time of the founding of the cancer registry in 1972: from >80 per 100,000 to ~20 in men by 2022 and from 22 to 6 in women (Figure 11). Historically, the major risk factors for liver cancer in Qidong have been infection with HBV (but not HCV), dietary exposures to aflatoxins, and the use of surface water for drinking [18,87]. Strategies, intended and serendipitous, have been directed at these three factors. A pilot program evaluating the safety and efficacy of a vaccine to prevent infection in newborns was undertaken in some Qidong townships from 1983–1990 [88]. Close to universal vaccination of newborns did not occur in China until the early 2000s. Consequently, most Qidong residents over the age of 40 have not been vaccinated against HBV. In 2021, the median age of diagnosis of liver cancer was 67 years (IQR: 57–74). Thus, the ~75% drop in liver cancer incidence has been driven by individuals never vaccinated against HBV. Infection rates have dropped dramatically in the birth cohorts that have been vaccinated, so that there is much anticipation for the impact of the vaccination program to be seen in the upcoming decades.

It has been proposed that the initial drop in age-standardized incidence rate from the 1970s into the 1980s reflects the success of a public health initiative to replace surface pond-ditch water with deep-well water as the primary source for all Qidong residents [87]. Deep-well water has been shown to have substantially lower levels of contamination by the blue-green algae toxin, microcystin. Microcystin is known to act as a hepatic tumor promoter in rodent models of carcinogenesis.

The increasing age of diagnosis of PLC in Qidong argues against advantages gained by improved healthcare in the region and benefits from screening programs. Indeed, survival rates following diagnosis have changed little over the past decades [11].

**Figure 12:**
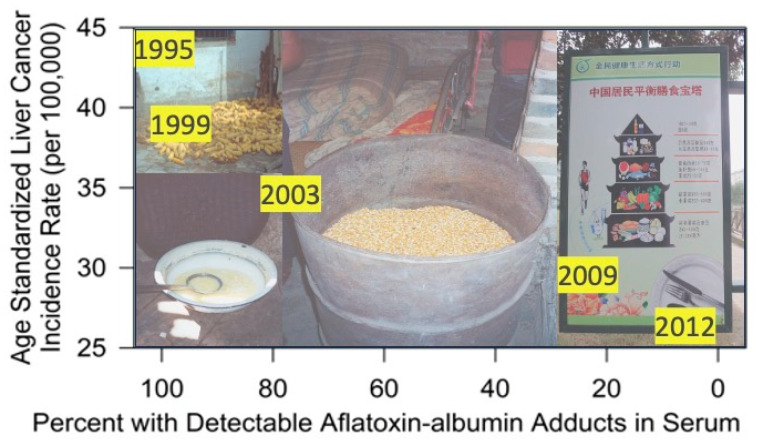
Decline in age-standardized incidence of liver cancer in Qidong with percentages of residents with detectable serum aflatoxin-albumin adducts collected between 1995 and 2012 (*N* = 100 for each year). Adapted from references [9,86]. *Inset:* drying corn, corn porridge, and household stored corn in rural Qidong (early 1990s) and a sign in a Qidong city park encouraging dietary diversity titled “Balanced Dietary Pagoda for Chinese Residents” (2012). Photo credits: Thomas Kensler.

By 2010, the signals from the Qidong Cancer Registry were quite strong for a robust and accelerating decline in age-standardized incidence of liver cancer. This observation prompted the QDLCI-JHU team to conduct a largely retrospective analysis of levels of aflatoxin albumin adducts in serum from samples archived from community screening programs and clinical trial eligibility screening [86]. As depicted in Figure 12, the percent of individuals with detectable levels of this aflatoxin exposure biomarker dropped from nearly 100% in 1995 to nearly undetectable in all samples collected in Daxin in 2012. All samples across the depicted series were age- and gender-matched. Over this 17-year period (1995–2012) the age-standardized incidence rate of liver cancer dropped from 44 per 100,000 in 1995 to 26 in 2012 when both genders are combined.

The driver for the dramatic drop in exposure to dietary aflatoxins was not through a directed public health initiative. Rather, it was the serendipitous result of economic reforms initiated by Deng Xiaoping and the Central Government in the late 1970s. Upon implementation in the mid-1980s, Qidong farmers could grow crops not just to meet local commune production quotas, but also to be sold in a broader marketplace for profit. Corn remained the primary crop in the region because of agricultural conditions unsuitable for rice cultivation, but rice became a common import from northern Jiangsu Province, whilst corn continued to be produced for use as animal feed and shipped out as an industrial raw material. Rice is far less susceptible to aflatoxin contamination than is corn (Table 1). By 1998, only 9% of families ate any corn, whilst the proportion of rural residents consuming some rice reached >99% around this period. Further, dietary diversity featuring many fruits, vegetables, and meats arrived with economic growth in the late 1990s and likely contributed to further reductions in aflatoxin exposures [89]. A market-driven eradication of aflatoxin from the diet was the key.

## 4. Epilogue

Field studies and clinical trials run by the QDLCI-JHU team ended in late 2019 with the onset of the COVID pandemic, border closures, and changes in international cancer funding priorities at the NIH. Over the 26 years of active, on-the-ground partnership, we conducted 10 clinical trials, enrolling ~1450 participants (even more people screened before entering the trials). With the recognition over a decade ago that aflatoxin exposures had been reduced many hundred-fold following market-based and other societal changes (Figure 12) [9,86] and with age standardized liver cancer rates beginning to follow (Figure 11) [11], our attention switched to lung cancer as an important emerging public health problem in the region (and indeed globally) [11,85,90]. Several randomized clinical trials, using ever-improving formulations of broccoli sprout beverages as the intervention agent, evaluated their efficacy to enhance the detoxication of toxic and carcinogenic ambient air pollutants such as benzene, acrolein, crotonaldehyde, and phenanthrene [75,76,77]. As seen in the earlier interventions focused on aflatoxin exposures, the goal was to enhance the detoxication of these pollutants. Indeed, greater than 60% increases in the rate of urinary elimination of benzene (as the benzene mercapturic acid) were observed in groups randomized to receive a broccoli-sprout beverage compared to a placebo beverage. These trials are the subject of several reviews [91,92,93,94].

Not only have disease focus and intervention agents evolved over the course of a quarter century of clinical trials in Qidong, but so have the biomarkers used as primary endpoints. Urinary biomarkers (aflatoxin metabolites and depurinated DNA adducts; air pollutant metabolites) have been used extensively in the Qidong studies. This biofluid is easy to obtain from participants, is generally not constrained by export regulations, and is relatively easy to clean up for analyses by mass spectroscopy. However, these biomarkers typically have short biological half-lives (<1 day), so they only reflect very recent exposures. They can be modulated quickly by some preventive interventions, offering a rapid read-out of efficacy. Longer half-life (~30 days) biomarkers such as carcinogen-albumin biomarkers offer the opportunity for integrated assessments of exposure, as in our study of the relationship of the declines in serum aflatoxin albumin levels and age-standardized incidence rates [54].

**Figure 13:**
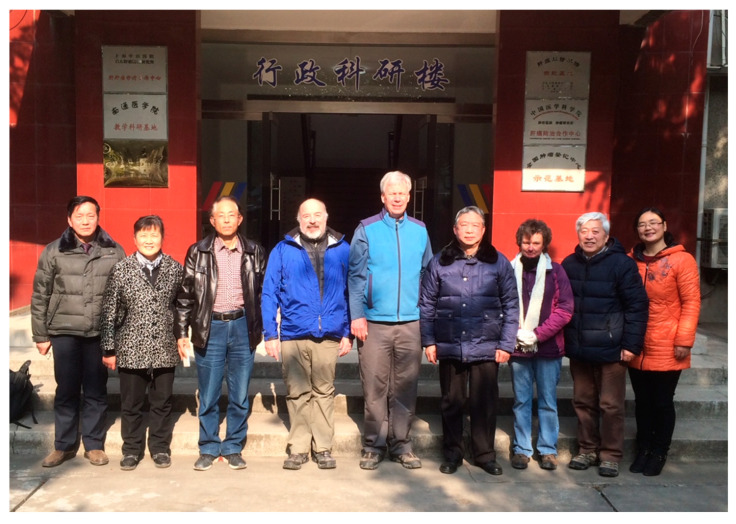
Last clinical trial team in Qidong (2018). The COVID pandemic and closed borders subverted ongoing and future field studies. (**Left** to **right**): Jin-Bing Wang, Jian-Hua Lu, Jian-Guo Chen, John Groopman, Thomas Kensler, Yuan-Rong Zhu, Patricia Egner, Geng-Sun Qian, Jian Zhu.

Analytically, only a few forms of aflatoxins need to be monitored given the dominant toxicities of AFB_1_ within the family of aflatoxins. Lysine is the primary, if not sole, amino acid for aflatoxin adduction in albumin. However, methods are being developed for multiplexing measures of environmental exposures within albumin. Many amino acid residues are known to be sites of covalent modifications by exogenous and endogenous electrophiles (principally Cys, but also His, Tyr, Ser, Met, and Arg in addition to Lys). Thus, albumin represents a stable macromolecular platform for discerning diverse exposures through the untargeted profiling of adducts. The Rappaport [95] and Groopman [96] labs, among others, have been leaders in the application of such proteomic technologies including data deconvolution in biomarker measures of complex exposures such as air pollution. Archived samples from the QDLCI-JHU repositories continue to provide substrates for new biomarker discovery and validation, although opportunities for further trials are not currently feasible in Qidong (Figure 13).

**Figure 14:**
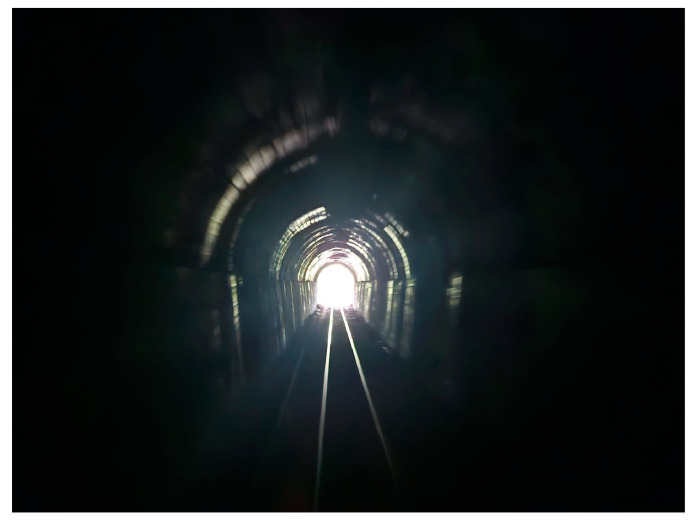
Using biomarkers and a cancer registry, Qidong research brings light to reduction of aflatoxin-induced liver cancer. Photo credit: Caroline Kensler.

The translation of the findings in Qidong regarding aflatoxins has important public health implications for the many countries worldwide whose residents are dependent upon a corn-based dietary staple. The capstone observation from Qidong is that primary prevention works—very effectively. Eliminate a dietary exposure, and health risks diminish. A change in diet from corn to a diverse menu of foodstuffs is not easily translatable globally. Corn is the leading grain harvested worldwide, and billions of individuals are potentially exposed daily to aflatoxin [4]. While regulators and policymakers worldwide have developed and implemented standards since the 1960s for aflatoxin contamination in foods and feeds with the goal of public health protection, better surveillance is required. Aflatoxin biomarkers such as the aflatoxin-albumin adduct allow for sensitive, high-throughput methods for surveillance within potential high-risk regions. Other prevention packages can be considered in addition to food monitoring, such as better storage practices for dietary staples, introduction of stress resistant-crops or non-toxigenic forms of *Aspergillus* into farmlands, or perhaps targeted chemopreventive interventions [4,97,98,99]. Partnering rigorous regulations or interventions with measurements of the sensitive and specific aflatoxin albumin biomarkers can complementarily and efficiently reveal the expected efficacy of these prevention measures [54] as projected by Professor Groopman in a 1999 commentary “The light at the end of the tunnel for chemical-specific biomarkers: daylight or headlight” [100] Figure 14.

## 5. The Essence of International Collaborations

There are several key elements for developing productive international collaborations that we have learned by trial and error and sometimes foresight. First and foremost is identifying the right people, followed by the development and nurture of relationships amongst the team. This takes effort. There must be a genuine interest in building friendships and cultural understanding. Investigators need to learn and experience each other’s cultures if the collaboration is going to be durable. It is important to identify and establish the commonality of goals and underlying motivations to achieve those goals. Some basic commonality of scientific training certainly facilitates these interactions, as does the discussion of the shared tenets of ethical and rigorous research and data management. Trans-national investigators should provide complementary sets of skills and resources to the projects, so that clear collaborative roles are evident to all. Diligence in maintaining regular channels for communication and meetings are important in all settings of collaboration. All parties should establish agreements/memorandums of understanding, which are often required by academic units and government-sponsored programs. These agreements should provide a framework for the governance of the research, the elements and expectations of funding, authorship, sharing of data and samples, and the intellectual property that may arise from the studies. The JHU investigators have collectively spent several decades of cumulative time “on the ground” in Qidong during the development, conduct, and renewal of our collaborative studies. Multiple reciprocal visits of Qidong scientists, clinicians, public health leaders, and government leaders to JHU have further nurtured the learning experiences and facilitated the timely conduct of the science.

Regulations at home and abroad govern the design and conduct of these collaborations. Meshing of regulatory and cultural distinctions between the collaborating countries can be challenging; this has certainly been true for our Sino-US projects, where the barriers have been bilateral. The Federalwide Assurance process, which defines the structure and functions of Institutional Review Boards, needed to be developed. Export and import of biological samples are subject to strict regulation in China, the US, and other countries. Transport can be very expensive; we bring samples with us on transoceanic flights to facilitate real-time conversations with customs officials should concerns arise. Solutions typically require creativity and capacity building in the country of origin of the biospecimens. Guidance on basic requirements must be available to all investigators. Cumbersome and protracted processes for securing permissions are an unfortunate hallmark of international collaborations. As an example, approvals for the 1995 oltipraz clinical trial were required from the IRBs of JHU and the QDLCI, the US NCI, the US State Department, the US FDA, the Shanghai Bureau of Public Health, and the Jiangsu Province Bureau of Public Health, as well as the Chinese Committee for the export of human biospecimens (the approval authority used to be the Ministry of Science and Technology, PRC, and now the National Health Commission, PRC), and the US CDC for their import.

It should be recognized that international partnerships in translational and other forms of research always takes much longer than anticipated. Passion and commitment to achieve the goal, along with patience, are essential prerequisites. Getting regulatory, infrastructure, and implementation factors synchronized is always underestimated in terms of scope and time. When international collaborative research works, the joy of new relationships, of building capacity, of making a difference in a place that has great needs, and of doing good science provides enormous satisfaction. The friendships that arise are enduring. This facet of collaboration is ever so evident in Qidong.

## Figures and Tables

**Table 1:** Aflatoxin B_1_ levels in grains collected in Qidong in three sampling periods. Adapted from [13].

Sampling Date	Grain Type	Samples Tested	AFB_1_ Positive	Positive Rate (%)	AFB_1_ Average (ppb)	AFB_1_ Range (ppb)
1972-Sept.	Corn	60	51	85.0		
	Wheat	33	8	24.2		
	Other	38	2	5.3		
	Total	131	61	46.6	182	5–4000
1973-May	Corn	176	65	36.9		
	Rice	29	0	0		
	Total	205	65	31.1	80.2	20–300
1973-Sept.	Corn	174	48	27.6		
	Rice	11	0	0.0		
	Total	185	48	25.9	113.8	5–1000

**Table 2:** Number of samples and detection rate of aflatoxin B_1_ content in Qidong corn. Adapted from [14].

Year	No. Samples	Detection Rate (%)	Aflatoxin B_1_ Content (%) *			
			<20 ppb	20–50 ppb	51–200 ppb	>200 ppb
1973	350	32.3	33 (29.2)	30 (26.6)	45 (39.8)	5 (4.4)
1974	522	25.5	66 (49.6)	53 (39.9)	11 (8.3)	3 (2.3)
1975	422	28.4	51 (42.5)	54 (45.0)	12 (10.0)	3 (2.5)
1976	359	50.4	91 (50.3)	68 (37.6)	20 (11.1)	2 (1.1)
1977	269	63.6	49 (28.7)	45 (26.3)	55 (32.2)	22(12.9)
1978	161	40.4	47 (72.3)	13 (20.0)	4 (6.2)	1 (1.5)
1979	106	33.0	27 (77.1)	5 (14.3)	3 (8.6)	-
1980	83	98.8	6 (7.3)	11 (13.4)	15 (18.3)	50 (61.0)

* The % in parentheses is the proportion of detectable positive samples.

**Table 3:** AFB_1_ levels (in ppb) in positive corn in Tongxing and Xining communes (1973–1975). Adapted from [15].

Commune	No. of Determinations	Within Permitted Level (<20)		Low Range (21–50)		Mid Range (51–250)		High Range (251–1000+)	
		No.	%	No.	%	No.	%	No.	%
Tongxing	96	34	35.4	49	51.0	10	10.4	3	3.1
Xining	24	17	70.8	5	20.8	2	8.3	-	-

**Table 4:** AFB_1_ intake and liver cancer mortality in Xining and Hehe communes. Adapted from [16].

Commune	AFB_1_ Intake (µg/Person/Day)	AFB_1_ Intake (µg/Person/Day/Weight)	Liver Cancer Mortality Rate per 100,000
Xining	0.0384	0.0006	14.47
Hehe	7.3638	0. 1273	53.37

**Table 5:** Summary of randomized clinical intervention trials using aflatoxin biomarkers in Qidong.

Agent (Year)	Dose and Schedule	Screening	Study Size (Duration)	Biomarker Modulation	References
Oltipraz (1995)	Placebo, *q.d*. 125 mg, *q.d.* 500 mg, *q.d.*	1006	234 (2 months)	2.6-fold increase in urinary excretion of AFB-NAC at 1 mo. (125 mg) and 51% decrease in AFM_1_ at 1 mo. (500 mg); 6% decrease in AFB-albumin adduct at 2 mo. (500 mg); no effect on urinary mutagens or oxidative DNA damage products	[52,53,54,55,82,83]
Chlorophyllin (1997)	Placebo, *q.d.* X3 100 mg, *q.d.* X3	511	180 (4 months)	55% decrease in urinary excretion of AFB-N^7^-Gua DNA adducts at 3 mo.	[66,67]
Glucoraphanin-rich (GRR) Broccoli sprout beverage (2003)	Placebo, *q.d.* 400 µmol GRR, *q.d.*	700	200 (14 days)	9% decrease in urinary excretion of AFB-N^7^-gua DNA adducts at 10 days; 10% decrease in air pollutant PheT excretion	[76]

## Data Availability

No new data were created or analyzed in this study. Data sharing is not applicable to this article.
